# Between and within-site variation in qualitative implementation research

**DOI:** 10.1186/1748-5908-8-4

**Published:** 2013-01-03

**Authors:** Justin K Benzer, Sarah Beehler, Irene E Cramer, David C Mohr, Martin P Charns, James F Burgess

**Affiliations:** 1 Department of Veterans Affairs, Center for Organization, Leadership, and Management Research, Boston, MA, USA; 2 Department of Health Policy and Management, Boston University, Boston, MA, USA

## Abstract

**Background:**

Multisite qualitative studies are challenging in part because decisions regarding within-site and between-site sampling must be made to reduce the complexity of data collection, but these decisions may have serious implications for analyses. There is not yet consensus on how to account for within-site and between-site variations in qualitative perceptions of the organizational context of interventions. The purpose of this study was to analyze variation in perceptions among key informants in order to demonstrate the importance of broad sampling for identifying both within-site and between-site implementation themes.

**Methods:**

Case studies of four sites were compared to identify differences in how Department of Veterans Affairs (VA) medical centers implemented a Primary Care/Mental Health Integration (PC/MHI) intervention. Qualitative analyses focused on between-profession variation in reported referral and implementation processes within and between sites.

**Results:**

Key informants identified co-location, the consultation-liaison service, space, access, and referral processes as important topics. Within-site themes revealed the importance of coordination, communication, and collaboration for implementing PC/MHI. The between-site theme indicated that the preexisting structure of mental healthcare influenced how PC/MHI was implemented at each site and that collaboration among both leaders and providers was critical to overcoming structural barriers.

**Conclusions:**

Within- and between-site variation in perceptions among key informants within different professions revealed barriers and facilitators to the implementation not available from a single source. Examples provide insight into implementation barriers for PC/MHI. Multisite implementation studies may benefit from intentionally eliciting and analyzing variation within and between sites. Suggestions for implementation research design are presented.

## Background

Qualitative research methods explicitly recognize that knowledge is incomplete, or ‘situated,’ and that analysis of multiple diverse perspectives across informants can enhance understanding of complex phenomena [1]. Studies of intervention implementation and organizational context vary widely in the analytical strategies used to account for this variation in perspectives. Some studies of implementation and organizational context incorporate multiple informants [2,3], whereas others rely on single-informant designs [4]. Some studies divorce respondents from their organizational context in order to summarize similarities in perspectives through either qualitative [5] or quantitative methods [6,7]. Some studies contrast the perspectives of key informants in analyses [8], but often the analysis of variation between informant types is not explicitly reported. Variation in perspectives among key informants may reflect substantive variation in how an intervention is implemented both within sites and between sites. Because perceptions can vary among key informants, understanding the nature and extent of this variation may be critical in determining why interventions succeed or fail. The purpose of this study was to demonstrate the importance of broad sampling in implementation studies by highlighting the role of qualitative variation in making inferences regarding both within-site and between-site implementation themes.

Most qualitative implementation research has one of three foci. First, some qualitative studies are conducted within the context of a randomized controlled trial [9,10]. Second, some qualitative studies investigate the factors that influence the spread of evidence-based practice across facilities or regions [11-13]. Third, some qualitative studies are designed to understand the factors that influence local adoption of evidence-based practices [6,14,15] and local tailoring of interventions [16]. Gathering rich data regarding how the organizational context affects the implementation process is important in all three areas [15,17]. Organizational context can affect factors such as implementation fidelity, processes of implementation, and intervention effectiveness. Qualitative research can be used to understand these factors in randomized controlled trials [18], studies of intervention spread [19], and adoption of evidence-based practices [20,21]. Research on the role of organizational context is particularly important in multisite implementation studies as local organizational conditions present different opportunities and challenges at each site.

Although the implementation science literature recognizes that organizational context impacts intervention implementation, there is not yet consensus on how to account qualitatively for within-site and between-site variation in organizational context. Many multisite implementation studies do not include qualitative methods [22-26], and those few that do tend to sample broadly across sites (and narrowly within a site) in order to understand between-site variation [27-29]. However, characteristics of informers, such as profession, leadership level, and role in the intervention, may influence perceptions of an intervention at a single site that may or may not generalize across multiple sites within the same study [30]. This variation is challenging for qualitative implementation research because it increases the cost and complexity of data analysis. To reduce this cost and complexity, many multisite qualitative implementation researchers make decisions that restrict sampling (*e.g.*, decreasing the length of engagement with a site or limiting sampling across different types of key informants) in order to manage the time and expense associated with multisite qualitative research. Restricted sampling in multisite qualitative implementation studies may be better than no multisite research at all, but it is important to carefully consider the tradeoffs researchers make between analytic and sampling strategies.

To facilitate the qualitative study of intervention implementation across sites, the authors developed a framework for analyzing within-site and between-site variation based on the literature on qualitative triangulation. The current study will apply this framework by analyzing the variation in how an intervention implementation was perceived by different sets of key informants within multiple sites. This may be particularly important in cases where the relevant implementation crosses professional boundaries, so we chose a multidisciplinary intervention that affects both primary care and mental health staff at a set of medical facilities. Results may be helpful to both qualitative researchers involved in multisite studies and intervention researchers who are required by funding agencies to include a qualitative evaluation component in multisite implementation studies.

## Methods

### Intervention

Primary Care/Mental Health Integration (PC/MHI) is a national effort in the Department of Veterans Affairs (VA) to improve the management of mental health in VA outpatient settings. PC/MHI was initiated in 2008 by a national mandate. Most PC/MHI programs (93%) include co-located mental health providers to increase the availability of mental health services in the primary care setting [31].

### Participant and clinic characteristics

We analyzed archival data drawn from a 2009 management evaluation that was designed to understand the processes used to provide co-located PC/MHI in a sample of hospital-based and affiliated freestanding outpatient clinics [32]. The VA Institutional Review Board approved these analyses. From the larger evaluation data set, we purposefully selected four clinics. Seventeen employees were interviewed in the selected clinics as part of the larger evaluation study. Clinic leaders and staff from both primary care and mental health were interviewed at each site. The four sampled sites were split between two different VA medical centers; each medical center is a quasi-independent facility with its own budget and management structure. Within each medical center, we selected one hospital-based primary care clinic and one large (10,000+ unique patients) community-based outpatient clinic (CBOC). The national PC/MHI mandate has similar standards for these large clinics. The four clinics in the current study were selected because the interviews demonstrated both within-site and between-site variation and because the affiliation between the hospital-based and freestanding outpatient clinics facilitates analysis of the variability within a single organizational authority structure. Thus, results will also show whether implementation factors varied across administratively connected sites. To protect confidentiality, sites were labeled for the current study as “Alpha,” “Bravo,” Yankee,” and “Zulu,” where Site Alpha was a hospital-based site affiliated with the same medical center as CBOC Bravo, and hospital-based Yankee was affiliated with CBOC Zulu. Key informants at each site included the specialty mental health leader (MHLs), staff providing PC/MHI care (PCMH), and primary care physicians (PCPs).

### Data collection

Semi-structured interviews were used to conduct a formative evaluation between July and August 2009. Formative evaluation was defined as an assessment of both potential and actual influences on implementation progress and effectiveness [33]. The purpose of the interviews was to understand how referral processes were implemented to coordinate mental healthcare between primary care and mental health staff. Stratified purposeful sampling was used to identify key informants [34]. We interviewed clinic leaders, who then identified staff who were the most knowledgeable regarding the PC/MHI implementation.

Interviews were collected from MHLs, PCMH, and PCPs. Telephone interviews were conducted for up to 45 minutes, with a note taker instructed to record responses verbatim where possible. To protect informant confidentiality, voice recording was not conducted. Data were collected until between-site saturation was reached. That is, saturation was judged to occur when interviews reported implementation issues that paralleled issues at other sites and no new implementation issues were reported. At most sites, interviews were conducted with each of two PCMHs and two PCPs. To protect confidentiality, and because the divergence within informant type (*e.g.*, between two physicians at one site) was not as striking as the divergence between informant type (*e.g.*, between PCMH and PCP at one site), within-type divergence will not be presented. In other words, we will present results from MHLs, PCMH, and PCPs, but not discuss variation within PCMH and within PCP responses. Instead, we focus on variation between positions and between sites. We will present results as if the PCMH and PC perceptions were each provided by one informant.

Using a case study model [35], an interview protocol was developed for the evaluation study that included open-ended questions that allowed the respondents to describe processes of care used in that site and to describe how those processes changed over time [36] with follow-up questions [37,38]. The evaluation team developed seven interview questions and specific probes to gather information on the processes used to coordinate primary care and mental health staff (see Table 1). Interviewers first asked two “grand tour” questions [39], which allowed informants to describe the present and evolved/past processes of care used in their sites openly and without a researcher-imposed framework. Interviews then turned toward interviewer-driven topics related to coordination, which was a focal point for the intervention and its evaluation.

**Table 1:** Interview questions and specific probes

**Interview question**	**Specific probes**
1. Imagine that a patient with depression symptoms comes to the clinic. Can you walk me through a typical process of care?	Referral process, differences between diagnoses
2. How has this process changed over the past 10 years (or since you arrived in the clinic)?	Recent changes, leadership support, referrals, interpersonal interactions, physical structure
3. Tell me about your sense of the need for coordination between primary care and mental health.	Examples of good and poor coordination
4. How would you change your clinic to better coordinate care?	Communication, collaboration, resource barriers
5. Have you or anyone you know had to develop your own coordination procedures to ensure that patients receive the best care?	Workarounds, ad-hoc coordination procedures
6. Can you tell me about the relationship between the people in the primary care and mental health clinics?	Face-to-face contact, trust
7. In what situations would you say that teamwork is most important?	Coworkers back each other up

### Triangulation of perceptual variation

Study analyses required researcher decisions regarding how to aggregate perceptions that were shared or unique. The purpose of these analyses was to identify common themes regarding the implementation (*e.g.*, leaders are important facilitators), while coding variation within these themes. Perceptions may therefore either converge toward these common themes or diverge toward a unique perspective [40].

Generally, the credibility of qualitative research can be established through prolonged engagement, persistent observation, and triangulation. [1]^a^Prolonged engagement (*i.e.*, research over an extended time period) provides a broad scope of understanding. Persistent observation (*i.e.*, focused investigation on specific topics) provides depth to the understanding of a specific phenomenon (Lincoln & Guba, p. 301–307). Triangulation involves a comparison of different kinds of evidence to evaluate the credibility of the evidence.

Triangulation used to support convergence across sources may be conceptualized as a validity test where perceptions that are not corroborated are seen as questionable. Convergence is often valued in implementation research because parallel perspectives across key informants can increase the validity of the data [41], but divergent perspectives are not necessarily invalid [42]. Triangulation may also be used to support divergence across sources by identifying patterns of distortion [43] common to particular sources and using those patterns to evaluate the credibility of the divergent perceptions. Few implementation studies that sample multiple key informants discuss the implications of divergence for intervention implementation, but differences between informant groups may be as important as similarities in understanding why an intervention succeeded, failed, or needed to be tailored for a specific site. Divergent perceptions indicate that an informant or group of informants interprets a shared phenomenon differently and can suggest the need for additional analyses, data collection, or follow-up clarifications to understand the meaning of the divergence. Failing to identify this variation in implementation studies may result in an incomplete understanding of the factors impacting intervention implementation. Convergent perceptions support understanding the multidimensional nature of implementation, whereas divergence can support rejection of a hypothesis, facilitate hypothesis construction, or highlight differences between sources [40].

### Analysis of within-site and between-site variation

The analytic strategy for the current study was developed following initial data analysis (for evaluation purposes) in which discrepancies across informants emerged. During that initial round of analyses, two coders sequentially analyzed the evaluation data, evaluated conceptual codes, revised codes, and resolved discrepancies [44]. During this analysis, we discovered substantial within-site and between-site variation in informants’ accounts of (1) the specific details of how co-located care was implemented and (2) the referral processes used to coordinate between primary care and mental health. The analyses for this study focused on exploring variation across informants in those two areas. Based on the initial data analysis, the authors collaboratively selected two medical centers to analyze more deeply (*i.e.*, the four sites selected for the current study). These sites demonstrated particularly clear examples of variation within and between sites. Data were then reanalyzed for the purpose of identifying both within-site and between-site implementation themes.

For the purposes of demonstrating how these analyses were conducted, we make a distinction between redundant, convergent, and divergent perceptions. Redundant perceptions provided information that was completely shared across informants (we note that complete agreement is a strict criterion adopted for the purpose of demonstrating what was truly shared across informants). Convergent perceptions provided information that elaborated redundant perceptions by demonstrating variation in interpretation of shared phenomena. For example, primary care and mental health may perceive different aspects of the same phenomenon. Finally, divergent perceptions reflected a unique departure from the convergent perceptions. Divergence suggests that a phenomenon is interpreted differently across sources.

Within each site, we analyzed the data from the perspectives of the three key informant groups to identify patterns of variation that distinguished one source from another [45]. One qualitative researcher categorized data as redundant, convergent, or divergent. Additional researchers then reviewed the transcripts and, through discussion, came to a consensus on the interpretation of the data. The researchers evaluated the credibility of divergent perceptions by analyzing both the pattern of responses within sites (*i.e.*, does the divergence fit with the responses of other informants) and also the patterns across sites (*e.g.*, does the divergence fit the types of responses from similar informants across sites). Within-site implementation themes were inferred from the convergence and divergence of responses among the key informant groups at a site. Between-site implementation themes were inferred from the patterns of responses across the four sites rather than simply comparing the different within-site themes. The analysis of convergent and divergent data used to identify implementation themes are described below.

## Results

Summary data are presented in Table 2 for Site Alpha, Table 3 for Site Bravo, and Table 4 for Site Yankee. Informants at Site Zulu provided unique, but not necessarily divergent, perceptions and are discussed separately. Tables present the redundant concepts at each site (*i.e.*, the descriptive data that were supported by perceptions from all three informants) and the convergent and divergent perceptions that elaborated the redundant concepts. The analyses below reflect the presentation in the tables. Redundant perceptions are described first, followed by the convergent and divergent perceptions. After the within-site themes are described at each site, we present an analysis of the between-site implementation theme.

**Table 2:** Convergence and divergence in referral procedures for Site Alpha

**Redundant concept**	**Convergence or divergence**	**Supporting data**
Psychologists are co-located in primary care	Convergence	MHL: Multiple revisions to service agreements were needed to tailor the implementation to primary care needs.
	Divergence	PCP: PC/MHI could be expanded to help manage difficult medical patients.
Traditional consultation-liaison service	Convergence	MHL: PC/MHI supplements the consultation-liaison (POD) services.
	Convergence	PCMH: POD services are separate from the implementation.
	Divergence	PCMH: POD restricts access to specialty mental health.
	Divergence	PCP: POD availability has improved since the implementation, but POD sometimes refers patients back to PC without treatment.
Implementation limited by a lack of space	Convergence	PCMH: Reported a growth in referrals since implementation and implemented innovative new procedures to make use of limited co-located staffing.
	Convergence	PCP: Acknowledged improved access, but doubted long-term impact due to lack of space.

Note: Supporting data are presented as either convergent or divergent with the redundant concept. *MHL* refers to the mental health leader at the site; *PCMH* refers to the Primary Care/Mental Health Integration (PC/MHI) informants at the site; PCP refers to the primary care physician informants at the site. *POD* = psychiatrist on duty.

**Table 3:** Convergence and divergence in referral procedures for Site Bravo

**Redundant concept**	**Convergence or divergence**	**Supporting data**
PC/MHI has improved access	Convergence	MHL: Co-location and the size of the clinic promote positive interactions, as providers see each other at lunch and at meetings.
	Convergence	MHL: PC/MHI goal is immediate access; PCMH is always available.
	Convergence	PCMH: Conducting a pilot study to provide access to walk-in patients.
	Divergence	PCMH: Getting buy-in from PC is biggest challenge; informal discussions in the lunch room and “selling ourselves” increased curbside consults.
	Divergence	PCP: Psychiatrists resisted helping PC manage behavioral aspects of chronic diseases, but negotiations have resulted in progress.
Referrals include standard referrals and curbside consults	Divergence	PCMH: Nurse routine screening often initiates referrals and some nurses refer patients inappropriately; working with nurse manager to educate staff.
	Convergence	PCP: Norms indicate that knocking on doors is appropriate, even if it interrupts ongoing psychological care.

Note: Supporting data are presented as either convergent or divergent with the redundant concept. *MHL* refers to the mental health leader at the site; *PCMH* refers to the Primary Care/Mental Health Integration (PC/MHI) informants at the site; *PCP* refers to the primary care physician informants at the site.

**Table 4:** Convergence and divergence in referral procedures for Site Yankee

**Redundant concept**	**Convergence or divergence**	**Supporting data**
Mental health providers are co-located in the primary care setting	Convergence	MHL: Co-location promotes communication and shared understanding, but lack of space limited the implementation.
	Convergence	PCMH: Only three primary care teams have co-located PCMH due to space restrictions.
	Divergence	PCP: Some primary care providers forced to give up space for PC/MHI—created conflicts; implementation planning was conducted unilaterally by MH.
PC/MHI team leader triages consults and assigns them to a PC/MHI provider	Convergence	PCMH: PC referrals to PCMH have increased, particularly for medical conditions such as diabetes management.
	Convergence	PCP: Implementation increased patient compliance with referrals.
	Divergence	PCP: PCMH helps refer appropriately, but some providers may not refer to PCMH at all.
	Divergence	PCP: Conflict between PCMH and ER regarding responsibility for urgent patients.

Note: Supporting data are presented as either convergent or divergent with the redundant concept. *MHL* refers to the mental health leader at the site; *PCMH* refers to the Primary Care/Mental Health Integration (PC/MHI) informants at the site; *PCP* refers to the primary care physician informants at the site. *ER* = emergency room.

Across the sites, we found that MHL reports generally agreed with PCMH and PCP reports, providing some support for the use of single-source MHL interview methodologies. The redundant concepts were particularly salient characteristics of PC/MHI implementation, but the aspects of these characteristics that were agreed upon were often of trivial importance to the implementation. Instead, the convergent and divergent perceptions of these redundant characteristics were critical to identifying implementation themes. PCMH informants provided the most detailed reports of the procedures, barriers, and facilitators for the implementation. This result was expected since they were the staff most directly tasked with implementation of the process. The perceptions provided by PCPs appeared to offer the most unique information, often capturing important contextual details that were not provided by other sources. Together, the perceptions gathered from these three sources (*i.e.*, MHL, PCMH, and PCP) facilitated site-level inferences about the implementation of PC/MHI that would not be available from any single source.

### Site Alpha

Three redundant PC/MHI concepts were identified at Site Alpha (Table 2). First, all informants agreed that the co-location of psychologists in the primary care setting was a key component of the PC/MHI intervention. Second, all informants acknowledged that the traditional consultation-liaison service was available; co-located psychologists were an additional mechanism for mental health access. Third, all informants reported that the PC/MHI intervention was limited by a lack of space.

### Psychologists are co-located

The MHL informant elaborated upon the common observation that psychologists are co-located by noting how the intervention required multiple revisions to the service agreements (*i.e.*, formal policies that standardize responsibilities between services) to fit with the needs of primary care. This convergent perception suggests the potential difficulties involved in adapting an intervention to existing organizational procedures. PCPs also acknowledged the co-located structure of PC/MHI, but generally expressed frustration with the intervention. PCPs noted that the goal of PC/MHI should be helping PCPs care for patients. PCPs suggested that PC/MHI would benefit from expanding to helping manage difficult medical patients, rather than focusing on specific mental health conditions. This divergent perspective indicates that the PCP and MHL representatives did not share the same opinion regarding the role of the co-located psychologist. That is, there was variation in whether informants felt that PC/MHI should be targeted specifically to mental health conditions or more broadly targeted. A PCP suggested that “old-fashioned team meetings” were needed to provide collaborative care for patients. This divergent perception reflects a general theme that cross-service collaboration was not part of the PC/MHI implementation at Site Alpha.

### Consultation-liaison service

The traditional psychiatrist on duty (POD) consultation-liaison service was mentioned by the three key informants, but perceptions of POD interactions varied greatly. MHL convergent perceptions suggested that PC/MHI supplemented the traditional consultation-liaison service by providing quick access for common mental health needs. PCMH agreed with MHL that the traditional POD consultation-liaison service was important for mental health access, but noted that PC/MHI services were not integrated with POD; rather, PC/MHI was a separate intervention added to the preexisting POD services. PCMH elaborated with a divergent perspective that the POD has a gatekeeper role and restricts, rather than facilitates, mental health access. PCP supported this perception and indicated that the POD referred patients back to PCP without treatment. This divergent perception suggests that the POD and PC/MHI were viewed as separate and potentially conflicting methods for facilitating mental healthcare in the primary care setting.

### Lack of space

Space was identified as a barrier by all three informants, but the effects varied. MHL reported space limited the number of co-located psychologists. PCP reported space created barriers to collaborative care and doubted the long-term impact of PC/MHI without collaboration. PCMH elaborated on the effect of the space limitation, reporting a growth in referrals since the implementation that created a screening bottleneck. Because no additional co-located staff was possible due to limited space, PCMH responded by developing innovative solutions to increase access with a minimal number of co-located psychologists. Specifically, PCMH tested a group screening procedure that decreased the time between referrals from PCP to PCMH to follow-up care (*e.g.*, POD, substance abuse, posttraumatic stress disorder referral). Thus, convergent perceptions suggested that the effectiveness of PC/MHI at Site Alpha may partially depend on local tailoring of procedures to overcome space barriers.

### Dominant implementation theme at Site Alpha

Variation in perceptions suggested a within-site theme that processes of coordination between services are critical to integrated care. To implement co-located care, PCMH reported engaging primary care, and PCP reported overcoming psychiatrist resistance. Regarding the consultation-liaison service, MHL indicated that the POD provided an alternative mechanism to access mental health services. Divergent perceptions elaborated that the POD was not well integrated with PC/MHI, and the POD process served a gatekeeper role conflicting with PC/MHI. The credibility of this conclusion was enhanced by both PCMH and PCP reporting that the preexisting role of psychiatrists/POD was a barrier to PC/MHI. Regarding lack of space, convergent perceptions from PCMH elaborated how innovative processes for mental health screening were developed on the frontline to handle high demand to overcome lack of space. Finally, there was divergence over the extent to which PC/MHI planning was one-sided, raising important questions about which types of patient care should be shared between primary care and mental health (*i.e.*, should PC/MHI address medical comorbidities?). Results suggested that the coordination processes, rather than structural factors (*i.e.*, co-location and space), facilitate and/or restrict PC/MHI implementation at Site Alpha.

### Site Bravo

Site Bravo (Table 3) is a community-based clinic that shares a common leadership structure with Site Alpha. Two redundant concepts were identified at Site Bravo. First, all informants agreed that the co-location of psychologists in the primary care setting improved access to mental health care. Before the implementation of PC/MHI, patients needing mental health treatment would travel more than an hour to Site Alpha for an appointment. Second, all informants reported that the PC/MHI intervention improved same-day mental health access though the processes of traditional referrals and “curbside consults.”

### PC/MHI improves access

MHL agreed that co-locating mental health providers next to primary care improved access. Convergent perceptions from MHL indicated that co-location and the size of the clinic promoted positive interactions, such as shared lunches and formal meetings. PCMH provided a divergent perspective for positive interactions and noted that getting “buy-in” from primary care providers was the biggest challenge. Contrary to the MHL perception that co-location and size had passive effects on referrals and curbside consults, PCMH actively used opportunities to “sell” themselves (*e.g.*, through informal discussions in the lunchroom). PCMH agreed that the goal of PC/MHI was immediate access and reported conducting a pilot study to provide access for walk-in patients. PCP provided a second divergent perspective, describing psychiatrists working in specialty mental health as one source of the resistance to PC/MHI implementation. According to PCP, the psychiatrists initially resisted helping PCPs manage the behavioral aspects of chronic diseases, but negotiations resulted in changes. Thus, several factors influenced access to PC/MHI care at Site Bravo, but perceptions across the three informants suggested that the implementation was dependent on inter-service collaboration.

### Same-day access

MHL provided a redundant perspective that the goal of PC/MHI was to provide immediate mental health access and that PCPs could choose to treat mental health conditions themselves or make formal referrals or informal “curbside consults.” PCP provided convergent perceptions that elaborated on the meaning of informal curbside consults (*e.g.*, knocking on a psychologist’s door for an informal consult that complemented formal consults). PCMH provided convergent perceptions that further elaborated same-day access to PC/MHI. PCMH reported that referrals often were initiated with nurse screenings; however, some nurses refer patients inappropriately. PCMH reported working with the nurse manager to educate staff to improve and create an appropriate referral processes. This convergent perception indicated that training may be a missing component of the intervention at some sites.

### Dominant implementation theme at Site Bravo

Variation in perceptions suggested a within-site theme that collaboration between services facilitated the implementation. MHL suggested that the structural aspect of the intervention (*i.e.*, co-location of mental health providers) facilitated access, whereas PCMH and PCP noted the importance of process factors (*e.g.*, active selling, psychiatrist resistance). In contrast to Site Alpha, these differences were attenuated by evidence of collaboration across services. PCP indicated that MHL was aware of their concerns, and primary care was either informed or involved with mental health planning (*i.e.*, negotiations). Mutual awareness suggests good communication regarding PC/MHI across the services. Collaboration was further evidenced by the interactions between PCP and MHL. Knocking on doors was an accepted method of receiving informal consults, and PCMH was comfortable providing feedback and training to PCP nurses regarding appropriate screening and referrals. Results suggest that collaboration across services facilitated implementation.

### Site Yankee

Two redundant concepts were identified at Site Yankee (Table 4). First, all informants agreed that the co-location of psychologists in the primary care setting was a key component of the PC/MHI intervention. Ten years prior to the interviews, Site Yankee had a co-located psychologist, but the growth of primary care created demands for space that resulted in the psychologist moving with the rest of mental health to a building one mile away. The integrated care mandate resulted in four rooms being allocated for two co-located psychologists and two psychiatrists to provide same-day consults to primary care patients for evaluation and short-term interventions. Second, all informants acknowledged that referrals were conducted through a triage system where the PC/MHI team leader assigned consults.

### Lack of space

All informants reported that space was a limitation, with PCMH indicating that only three primary care teams within the site had co-located mental health providers. In contrast to MHL and PCMH, who each indicated space was a structural barrier that limited full staffing for the intervention, PCP reported a severe conflict between primary care and mental health because some PCPs were forced to give up their space. PCP attributed the problem to MHL unilaterally planning for implementation. This divergence between informants suggests a poor collaboration between primary care and mental health that may pose a serious threat to implementation sustainability.

### Triage referral system

All informants reported that Site Yankee used a triage system for assigning patients to mental health providers. PCMH elaborated the referral process, suggesting that an increase in referrals in recent months, particularly for medical conditions such as diabetes, was a sign of implementation success. PCP complemented the PCMH view that the implementation was successful, but suggested that the reason for this success was increased patient follow-up on mental health referrals. Further, PCP suggested intervention spread was a concern at Site Alpha. PCP reported that PC/MHI providers were valuable in helping PCPs navigate the extensive mental health treatment options at the site, though some PCPs may not have referred to PC/MHI at all. PCP also reported divergent perceptions regarding a conflict between the PC/MHI providers and the emergency room (ER). According to PCP, ER perceived that mental health urgent care should be the responsibility of PC/MHI, whereas PC/MHI felt that these patients should still be treated in ER. This conflict was resolved with assistance from leadership after a period of referring patients back and forth to PC/MHI and the ER. The ER example indicates that the PC/MHI intervention may conflict with existing procedures, and leaders should be aware of this potential and be ready to resolve conflicts.

### Dominant implementation theme at Site Yankee

Variation in perceptions suggests a within-site theme that poor collaboration between primary care and mental health leaders has limited the implementation. In particular, PCP divergent reports regarding the conflict over space suggests a lack of collaborative planning for the PC/MHI implementation, which may have slowed intervention spread through primary care. Similar divergences between PCP and MHL regarding a lack of collaborative planning were evidenced at Site Alpha, suggesting a pattern of divergence between primary care and mental health across sites. Conflict over space is a particular concern for intervention sustainability at Site Yankee, given that a co-located care program was ended 10 years previously due to a lack of space. A lack of collaboration between primary care and mental health was further evidenced by differences in the perceived success of PC/MHI. The conclusion that Site Yankee implementation was successful implementation is supported by the convergence between PCMH and PCP, but interviewing multiple informants provided a richer understanding of the boundaries of this success. For example, the evidence for success from the PCP perspective might be characterized as patient-centered (*e.g.*, increased patient follow-up), whereas evidence from the PCMH perspective might be characterized as provider-centered (*e.g.*, increased referrals). These results support a broad within-site implementation theme of poor collaboration between primary care and mental health.

### Site Zulu

Site Zulu is a community-based clinic that shares a common leadership structure with Site Yankee. Primary care and mental health had previously been located in the same building, but increased staffing related to the implementation of PC/MHI created space restrictions that were solved by moving all non-medicating PC/MHI providers to a separate building over a mile away. Psychiatrists are still co-located in primary care to provide medication support to primary care physicians. PC/MHI implementation is based on electronic referrals. Referrals are sent to the mental health nurse practitioner, who triages and assigns them to a mental health provider. Communication back to primary care is done through additional signers on the electronic medical record. Informants agreed that the implementation did not change the referral procedures. Rather, the key aspect of the intervention was reported to be the increased staffing that speeds the referral process.

### Unique perceptions

Site Zulu was unique among the sampled sites due to geographical separation between primary care and the non-medicating providers. Redundant perceptions were common among informants. A limited amount of convergent information elaborating referral processes was collected at this site, but the information lacked a common theme. MHL stated that local VA regulations had previously required mental health providers to approve psychiatric medications, but procedures had been changed to allow primary care physicians more responsibility. PCMH was the only informant to note that patients can be discharged from the hospital with PC/MHI appointments, a valuable process for post-discharge continuity of care. PCP revealed a coordination problem involving psychiatric admissions. Local police would not escort patients to the psychiatric hospital because it is located in a separate county, and procedures were developed where a psychiatric nurse would escort patients to the hospital.

### Dominant implementation theme at Site Zulu

Site Zulu presents a unique model of PC/MHI compared to the other sites. Co-location was limited to psychiatrist medication support for PCPs, and therapy was provided through what was essentially a specialty mental health clinic focused on primary care concerns. Convergence of perspectives at Site Zulu was much stronger than for other sites; all key informants agreed on the basic referral process. Unique information was presented by each key informant, with each providing information specific to their domain of patient care.

### Variation between sites

Key informants across the four sites described implementation resistance and space limitations as common barriers to PC/MHI implementation, although this is not particularly informative for understanding how to facilitate the intervention. A cursory review of these themes might suggest a common theme that coordination, collaboration, and communication are important factors in PC/MHI implementation when sites choose to co-locate therapists in the primary care setting. However, analysis across sites revealed a between-site implementation theme that is more informative regarding the PC/MHI implementation. Between sites, the variation suggests that the preexisting structure of mental health care influenced how PC/MHI was implemented at each site. Further, communication and collaboration among both leaders and providers was critical to overcoming structural barriers that arose during PC/MHI implementation. The empirical support for both the within-site and between-site themes is presented in Table 5.

**Table 5:** Empirical support for within-site and between-site implementation themes

**Site**	**Within-site theme**	**Support for within-site theme**	**Support for between-site theme**
Alpha	Coordination processes between services rather than structural factors required for implementation	· Co-located structure present, but limited cross-service collaboration	· Space and preexisting consultation-liaison agreements are structural barriers
		· POD and PC/MHI viewed as separate mechanisms for mental health access	· Neither barrier resolved by collaboration; space resolved through PCMH innovation
		· Local tailoring of processes addressed structural space barrier	
Bravo	Communication and collaboration facilitated PC/MHI implementation	· Inter-service collaboration resolved differences	· Mutual awareness of concerns between services
		· Open communication facilitates patient access and process improvement	· Similar barriers to Site Alpha, but in Site Bravo, barriers were resolved through negotiation
Yankee	Poor collaboration between primary care and mental health caused implementation problems	· Space conflict	· Space and ER coverage agreements are structural barriers
		· ER referral procedure conflict	· Lack of collaboration appears to lead to conflicts over structural differences
		· Different definitions of intervention success	
Zulu	Prior failure implementing co-located care influenced decision to physically separate services	· MH leader report of prior failure	· Prior space limitation influenced implementation
		· No divergent perspectives of shared phenomena, possibly due to physical separation between PC and MH	· PC/MHI adapted to preexisting space barrier

Note: Between-site implementation theme: pre-existing structure influenced how PC/MHI was implemented; collaboration and cooperation among leaders and providers helped overcome these structural implementation barriers. *POD* = Psychiatrist on duty; *PC/MHI* = Primary Care/Mental Health Integration; *ER* = emergency room; *PC* = primary care; *MH* = mental health.

PC/MHI implementation was characterized by conflicts between primary care and mental health, but communication and collaboration between primary care and mental health were found to facilitate the implementation. As one example, psychiatrist resistance to the intervention was reported by primary care and the co-located mental health clinicians at both Site Alpha and Site Bravo. This resistance appeared to be due in part to how patient care was divided between primary care and mental health. Site Alpha faced challenges adjusting the formal agreements between the services, and Site Bravo encountered similar resistance in the physician suggestions to involve mental health in the care of medical illness. A key difference is that the Site Bravo MHL appeared to be responsive to PCP concerns, as negotiations were reported to result in tailored changes to the intervention implementation. In contrast, Site Alpha was characterized by one-sided planning and poor communication between services, which limited collaborative care. Site Yankee reported a similar pattern of implementation as Site Alpha. These two sites are connected only loosely by their shared administrative structure. Thus, the one-sided planning at Site Yankee suggests that this style of implementation may have been common in the PC/MHI intervention and appears to have caused some potentially preventable implementation problems.

Available space was a structural barrier to PC/MHI implementation. Leadership responses to these barriers at Sites Yankee and Zulu may have exacerbated implementation problems. Yankee leaders appear to have forced the implementation despite these constraints, whereas Zulu leaders chose to only co-locate psychiatrists to provide medication support to primary care and otherwise retained their model of referring patients to a geographically separated annex for therapy. In contrast, at Site Alpha a psychologist developed group screening procedures to meet the high demand for mental healthcare. Referral processes were also structural barriers to PC/MHI implementation at Site Alpha (*i.e.*, POD referrals), Bravo (*i.e.*, degree to which PC/MHI should be concerned with medical issues), and Site Yankee (*i.e.*, ER referrals). Differences between primary care and mental health were reported to have been resolved through collaboration among leaders at Site Bravo, and leaders resolved the ER conflict at Site Yankee. Thus, managing preexisting structural conditions in Sites Alpha, Yankee, and Zulu was a key difference between sites that appeared to affect how PC/MHI was implemented.

## Discussion

The current study demonstrates the value of analyzing both between-site and within-site variation among sources in qualitative implementation research. Analysis of variation across key informants facilitated the identification of between-site themes that the preexisting structure of mental healthcare influenced how PC/MHI was implemented at each site and that collaboration among both leaders and providers were critical to overcoming structural barriers. Results suggest that if interviews had only been conducted with MHLs, then those perceptions would describe the broad characteristics of the intervention, but those interviews would provide less detail regarding the specific processes implemented, or the barriers to that implementation. Interviews with frontline staff revealed hidden conflicts and differences in interpretations of the same intervention. The analysis of qualitative data from multiple perspectives complicates and enriches studies of multisite intervention implementation and contributes to the implementation science literature in two ways. First, the examples of discrepancies between primary care and mental health provide a concrete illustration of the practical implications of identifying both within-site and between-site implementation themes. Second, results have methodological implications for conceptualizing multisite implementation science research.

### Implications for implementation science

Conclusions regarding the PC/MHI implementation can vary depending on whether data are analyzed within a site or across sites. Analyzing variation both within and across sites may reveal generalizable patterns of variation. For example, each implementation was shown to be sensitive to the degree to which the intervention focused on issues related to medical conditions and the processes used to provide same-day urgent mental health access. Analyses within and across sites may also reveal variation in how the common barriers impacted implementations. For example, space was a barrier in all four sites, but the impact of space on the implementation varied across sites, and perceived implications of space constraints tended to vary within site across the key informants. Finally, analysis across sites may highlight some important site-specific issues with PC/MHI implementation that may or may not generalize to other sites. For example, the distinction between a provider-centered and patient-centered perspective of intervention success was only revealed at Site Yankee, but the differences between informant perspectives may have occurred at other sites. Certainly, it is reasonable to expect that informants who are directly involved in an implementation are likely to view changes in processes as a success, whereas those who are outside the intervention may be more likely to expect tangible impacts on patient care, but additional data collection is needed to confirm this difference.

Results indicated that administrative connections between medical centers and community-based clinics accounted for minimal variation in implementation. Although there were some similarities between administratively connected sites (*e.g.*, the practice of using lunch meetings to increase PCP buy-in was adopted at both Site Alpha and Site Bravo), results indicate that researchers should not assume that interventions are implemented the same in administratively connected sites. For example, in Site Alpha, the POD was considered outside the scope of the PC/MHI intervention, but the POD procedures were reported to affect the ability of PCMH providers to consult with specialty mental health providers. The POD procedures were a key feature of the organizational context for the intervention at this site. In contrast, psychiatrist resistance at Site Bravo was reported to be reduced through inter-service negotiation. The variation between these two administratively interconnected sites is likely due in part to differences in organization and mission between hospital-based and community-based outpatient clinics, but more broadly, history and preexisting processes used to provide mental healthcare at each site appeared to cause variation in the implementation barriers. As another example, both Site Zulu and their parent facility Site Yankee had previously implemented co-located care, but space limitations led to abandonment of that model. Site Yankee chose to attempt to reintegrate mental health staff into the primary care setting, whereas Site Zulu chose instead to capitalize on the preexisting referral processes. For medication support, Site Zulu provides limited access to specialty care psychiatrists already located in the same building. For therapy, Site Zulu continues to refer patients to a distant annex. Given the implementation difficulties at other sites, building on existing processes rather than attempting process redesign could be an effective strategy for improving patient access to mental healthcare.

The current study also highlights an important issue when considering the fit of an intervention with a specific organizational context. Implementation science has long recognized that misfit can be solved by either modifying existing practices or modifying interventions [46]. Three of the four sites chose to modify existing practices, but Site Zulu chose instead to modify the intervention by providing limited co-located mental healthcare through medication support and use the intervention funding to expand their existing mental health services. Given that space restrictions led to prior efforts at Site Zulu to provide co-located care to fail, this choice is certainly rational. Indeed, the goals of the PC/MHI intervention (*e.g.*, improving access to mental health services) may be more easily achieved by working within the parameters of existing procedures rather than attempting large-scale organizational changes that conflict with local operating conditions (*e.g.*, implementing co-located care in a site without the necessary space). However, the benefits and tradeoffs in this type of intervention modification are not clear in the current study. This may be a fruitful area for future research to help plan more effective inter-service interventions.

### Methodological implications

The key informant interview is a ubiquitous methodology in implementation science, but sampling for multisite qualitative implementation studies can be more complex than in other qualitative health research. Qualitative theoretical sampling aimed at revealing grounded theory is typically conducted until thematic saturation is reached, that is, until no new information is discovered [44]. However, the current study indicates that sampling can be complex in multisite implementation studies with multidisciplinary workgroups because variation may occur across professions and positions within site and between sites. Thus, the meaning of saturation may change depending on the populations being considered. Implementation study designs should consider the implications of these variations for generalizability of results and validity of conclusions.

Conceptual frameworks guiding qualitative implementation research should be explicit regarding variation among informants so readers can gauge initial standpoints and assumptions of the researchers (*e.g.*, the degree to which researchers allowed for different types of converging/diverging perceptions to surface). Building implementation theories on convergent perceptions can limit the practical utility of those theories in accounting for divergent factors, such as differences in perceived responsibilities and unintended consequences of interventions. The current study provides concrete examples of how and why key informants may perceive different aspects of an intervention. For example, convergent perceptions from mental health focused on how the organization structure affected processes (*e.g.*, co-location promotes positive interactions). In contrast, both PCMH and PCP were able to provide detail regarding the specific processes used to coordinate patient care. PCMH identified specific structural barriers to the implementation (*e.g.*, limited implementation of co-location, lack of education in nursing and clerical staff). PCP reported unique details regarding the implemented practices and outcomes (*e.g.*, can knock on PCMH door if needed, patient referral compliance has improved). Without these multiple frontline perspectives at each site, neither the within-site nor the between-site implementation themes could have been identified.

It is important that implementation researchers understand their role in processing and presenting divergent perceptions [43]. Divergence can be as important as convergence in understanding a phenomenon, but the uniqueness of divergent perceptions may cause some to question their validity [42]. Multisite studies may present specific concerns regarding the credibility of divergent perceptions because within-site sampling is likely to be restricted in order to sample across sites. However, multisite studies may have a unique advantage in the analysis of divergent perceptions. The current study established the credibility of divergent perceptions by identifying consistent patterns of distorted perceptions across the multidisciplinary workgroups within a specific source [45].

We propose that distortions in PC/MHI-related perceptions were derived from differences in key informants’ work roles. For example, the PCP and MHL disagreed regarding the key barriers to implementation in Site Alpha. PCP described the lack of collaboration from psychiatrists, whereas PCMH reported that getting buy-in from PCPs was the biggest challenge. As another example at Site Yankee, the PCP and MHL disagreed regarding the definition of implementation success. PCMH believed that intervention success was indicated by increased referrals from PCPs, whereas the PCP believed that intervention success was indicated by increased patient compliance. Conclusions based on interviews with only limited types of frontline staff in multidisciplinary work environments may be biased toward the perspective of those positions (or other relevant characteristics). Thus, while multisite implementation studies can be conducted with a limited sample of organizational positions, the validity of the conclusions based on the data may be limited. Implementation researchers should consider the benefits and tradeoffs of cost-effectiveness versus comprehensiveness regarding collecting data from multiple positions in their studies. The current study indicates that complete coverage of the key multidisciplinary positions impacted by the intervention (*e.g.*, primary care and co-located mental health frontline staff) is needed to minimize bias in conclusions made by researchers regarding the success of implementation.

Methodologically, the current study affirms the value of concurrent data collection and analysis during implementation research [47]. Perceptions from one source can be compared with other sources, and divergent perceptions should be carefully probed during data collection to understand why these factors are not shared among informants, whether there are systematic patterns of divergence, and/or why informants would fail to report the divergent factors. For example, the unilateral intervention planning by mental health was noted as a barrier at Sites Alpha, Bravo, and Yankee, but was only revealed in front-line interviews. Site Alpha only had one co-located psychologist. If this report was not corroborated by the primary care interviews, then credibility would be questionable in a single-site study. However, the pattern of responses across sites suggests that unilateral planning by mental health was a common practice in PC/MHI implementation. In this example, further data collection could have been conducted at Site Yankee and the other sites to understand why MHLs did or did not involve PCPs in their implementation planning.

In the qualitative study design, researchers should build in time for discussion and reflection on convergent and divergent perceptions between interviews. This would provide opportunities for implementation researchers to revise sampling strategies and interview questions as seemingly relevant patterns of divergence emerge. Although these types of designs require significant commitment during the data collection process, we suggest that they are cost effective in that these designs may greatly enhance the effectiveness of implementation research.

### Limitations

There were three main limitations to the current study. First, as the data collection was part of a multisite evaluation, the number of informants at a single site was necessarily limited. The current study did not analyze variation within employee positions. For example, the sampling was designed to identify the most knowledgeable informants, but interviewing less engaged primary care staff could have been valuable to further probe discrepancies between primary care and mental health. It is possible that within-informant differences could impact perceptions of the intervention, and potentially impact the utility of conclusions. Second, these sites were purposefully sampled from the evaluation sites because initial data analysis indicated both convergence and divergence. These are four case studies and may not be typical of PC/MHI intervention implementations in the VA. The sites were selected to provide an illustrative example of the importance of reporting both convergent and divergent perceptions in implementation studies. Third, this study was based on archival data. There was no opportunity to collect additional data to clarify divergent perceptions, but we acknowledge that additional data collection would have been valuable in exploring the discrepancies identified in the current study. It is our experience that this type of design is common in the practice of intervention evaluation. Studying perceptual divergence may require modification to study designs to promote concurrent analysis. Rapid analysis after data collection can identify divergent perceptions that may be probed in follow-up interviews. This type of design is common in longitudinal qualitative methods, such as ethnographies, but may also be critically important in implementation research. Evaluation designs that include planned follow-up interviews should be promoted by both researchers and funding agencies, but if funding does not allow for follow-up interviews, at the very least, implementation researchers should be sure to collect perceptions from all of the relevant stakeholders who are impacted by an intervention.

## Conclusion

The current study demonstrated that attention toward variation across organizational positions and sites can enrich implementation research by illustrating how shared themes are perceived by different stakeholders. This variation may be important when replicating interventions as a deep understanding of implementation factors may be needed to align interventions with varying stakeholder needs. Divergent perceptions were found to be potentially important information for understanding why an implementation may or may not succeed. Given the time and funding limitations inherent in implementation research, it is important to emphasize the benefits and design implications inherent in analyzing divergent perceptions in order to increase the quality and effectiveness of implementation studies.

## Endnote

^a^Triangulation can be used to resolve variation among sources, methods, investigators, or theories, but this paper is concerned with the most common use, triangulation among sources.

## Competing interests

The authors declare that they have no competing interests.

## Authors’ contributions

JKB, IEC, DCM, and JFB conducted the data collection and analyses. All authors contributed to writing the manuscript. All authors read and approved the final manuscript.
